# Dilemma of zebras: an unusual case of Hemophagocytic Lymphohistiocytosis

**DOI:** 10.1186/1710-1492-8-S1-A16

**Published:** 2012-11-02

**Authors:** Tahira Batool, Michael Cyr

**Affiliations:** 1 Dept. of Clinical Immunology and Allergy, McMaster University, Hamilton, ON, Canada

## Introduction

Hemophagocytic Lymphohistiocytosis (HLH) is a rare histiocyte disorder associated with perforin-dependent cytotoxic function, characterized by a highly stimulated, but ineffective, immune response to antigens, which results in life-threatening cytokine storm and inflammatory reaction. Both familial and secondary forms have been described. Secondary HLH in associated with infections, malignancies and rheumatological disorders.

## Case description

A 26 years old male with past medical history significant for neurosarcoidosis, presented with fever, dyspnea and right upper quadrant pain. He developed respiratory failure and required intubation within 48 hours. He was found to have profound pancytopenia (Hb 66, Platelets 4, WBC 0.7, neutrophils 0.1), hepatosplenomegaly, elevated liver enzymes, CK, ferritin (13080 microgram/l), triglycerides (6.3 mmol/l), low fibrinogen (1.4 g/dl) along with CNS lesions, lung infiltrates, proteinuria, renal failure. He had absent NK cell activity but no evidence of hemophagocytosis on his bone marrow or lymph node biopsy. He was treated as sepsis with no improvement. A trial of high dose corticosteroids and Anakinra (Anti IL-1) failed. His clinical condition deteriorated rapidly and he died after episode of massive pulmonary hemorrhage.

## Discussion

Secondary HLH is considered less common then familial forms. Although number of studies have suggested higher prevalence, it remains under recognized due to clinical similarities to severe sepsis making diagnosis difficult. HLH is an important consideration for critically ill patients not responding to conventional therapy.

